# Beyond counting intended pregnancies among young women to understanding their associated factors in sub-Saharan Africa

**DOI:** 10.1093/inthealth/ihab056

**Published:** 2021-09-14

**Authors:** Luchuo Engelbert Bain, Bright Opoku Ahinkorah, Abdul-Aziz Seidu, Eugene Budu, Joshua Okyere, Eugene Kongnyuy

**Affiliations:** Lincoln International Institute for Rural Health, College of Social Science, University of Lincoln. Brayford Pool, Lincoln, Lincolnshire. LN6 7TS, UK; Global South Health Research and Services, GSHS, Amsterdam, The Netherlands; School of Public Health, Faculty of Health, University of Technology Sydney, Sydney, NSW, Australia; Department of Population and Health, University of Cape Coast, Cape Coast, Ghana; College of Public Health, Medical and Veterinary Sciences, James Cook University, Australia; Department of Estate Management, Takoradi Technical University, Takoradi, Ghana; School of Public Health, Faculty of Health, University of Technology Sydney, Sydney, NSW, Australia; Department of Population and Health, University of Cape Coast, Cape Coast, Ghana; United Nations Population Fund, UNFPA, Bamako, Mali

**Keywords:** DHS, intended pregnancies, public health, sub-Saharan Africa, young women

## Abstract

**Background:**

In this article we report the prevalence and determinants of intended or wanted pregnancies among young women 15–24 y of age in selected sub-Saharan African countries.

**Methods:**

This cross-sectional study used pooled data from current Demographic and Health Surveys conducted between 1 January 2010 and 31 December 2019 in 29 countries in sub-Saharan Africa (SSA). The sample size comprised 14 257 young women (15–24 y of age). Multivariable binary logistic regression models were used to present the results as adjusted odds ratios.

**Results:**

The prevalence of intended pregnancies was 67.7%, with the highest and lowest prevalence in Gambia (89.9%) and Namibia (37.7%), respectively. Intended pregnancy was lower among young women who had knowledge of modern contraceptives, those with a secondary/higher education and those with four or more births. Lower odds of intended pregnancy were observed among young women in the richer wealth quintile and those who lived in southern Africa.

**Conclusions:**

To reduce intended pregnancies in sub-Saharan African countries such as Gambia, Burkina Faso and Nigeria, there is a need for government and non-governmental organisations to recalibrate current and past interventions such as investment in increasing formal education for women and poverty alleviation programmes, as well as augmenting job creation, including skill-building. These interventions have to be sensitive to the cultural realities of each setting, especially with regards to early marriages and womanhood.

## Introduction

More than 1 million adolescent girls ages 10–14 y and an estimated 16 million ages 15–19 y give birth each year.^1^ About one-third (29–33%) of pregnancies in sub-Saharan Africa (SSA) have been described as unintended: mistimed (occurring when a woman did not want to become pregnant at the time of pregnancy) or unwanted.^1–3^ More than 50% of unintended pregnancies end up as abortions and 97% of these in Africa are classified as unsafe.^1^ In Namibia for instance, more than 50% of pregnancies are unintended.^3,4^ The World Health Organization (WHO) reports that >12 million women ages 15–19 y and at least 777 000 girls <15 y of age give birth each year in low- and middle-income countries (LMICs).^1^ In a systematic review and meta-analysis carried out in 2018, the prevalence of adolescent pregnancy in SSA stood at 19.3%.^5^

Adolescent pregnancy carries a higher risk of pregnancy- and childbirth-related complications such as low birthweight, pre-eclampsia/eclampsia, preterm delivery and maternal and perinatal mortality.^1^,^6–8^ Adolescents are often physiologically and anatomically immature, which predisposes them to more incidents of difficult deliveries (caesarean sections, instrumental deliveries) compared with younger adults.^1^ Pregnancy- and childbirth-related complications are the leading causes of death among adolescents between 15 and 19 y of age in LMICs.^1^ Moreover, adolescent pregnancy is associated with low educational attainment, especially in LMICs. Indeed, the offspring of adolescent pregnancies are more likely to become teenage parents as well, thus causing a generational cycle of people at social and economic risk.^1,6^ Adolescent mothers also have diminished socio-economic status, affecting both the children and their mothers.^6,7^

In a recent systematic review on the prevalence and determinants of adolescent pregnancy in SSA, Yakubu and Salisu^4^ reported a series of sociocultural, environmental, economic, individual and health service–related factors. Most pregnancies among adolescents have been reported as more likely to be unintended.^2,3^ Considering the greater negative public health impact of unintended pregnancies among adolescents (high unsafe abortion-associated burden), a lot of attention has focused on unintended pregnancy, especially among adolescents. Not all adolescent pregnancies are unintended. Some reasons cited by adolescents for intentionally getting pregnant include to be like their peers, to gain respect in the community, to receive financial support from their partner and pressure from their parents.^9,10^ Early marriages in some communities render categorization of early adolescent pregnancy (intended or unintended) difficult. Indeed, the institution of marriage dilutes the voice of the adolescent in deciding upon when to get pregnant, and there is a greater likelihood of pregnancies in marriage to be classified as intended.^9–11^

Due to its high prevalence, most studies and interventions have focused mainly on unintended pregnancy. However, it is important to understand the drivers of intended pregnancy among young people. Indeed, it is possible to uncover some misconceptions. Postponing or preventing pregnancies among adolescents will not only avert the health-related consequences associated with adolescent pregnancy but will allow these adolescents to stay in school longer and lead more economically productive lives. This is in line with the Sustainable Development Goals (SDGs) on good health and well-being (goal 3), quality education (goal 4) and decent work and economic growth (goal 8).^12^ Pregnancy and childbirth (intended and unintended) are the major causes of deaths among adolescents.^1^ Interventions should therefore tackle the drivers of both unintended as well as wanted adolescent pregnancy. The literature on the determinants of pregnancy among young women in SSA is sparse. Measuring intended pregnancy rate and its determinants among adolescents (15–19 y) and young adults (20–24 y) is relevant in setting up age-specific interventions. We aim to report the prevalence and determinants of intended or wanted pregnancies among young women ages 15–24 y in selected SSA countries.

## Methods

### Study design and data source

This was a cross-sectional study that used pooled data from current Demographic and Health Surveys (DHS) conducted between 1 January 2010 and 31 December 2019 in 29 countries in SSA. These countries were included in the study because their surveys had information on all the variables of interest. The DHS is a nationwide survey executed every 5 y across LMICs and is representative of each of the countries. Files that have responses from women ages 15–49 y were used in the study. The surveys targeted core maternal and child health indicators, including unintended pregnancy, contraceptive use, skilled birth attendance, immunisation of children <5 y of age and intimate partner violence. A stratified dual-stage sampling approach was employed and the same questions were posed to women in all these countries and thus makes it feasible for multicountry studies. The study involved a multistage sampling process (i.e. enumeration areas [EAs]), followed by systematic household sampling within the selected EAs. The sample size for this study comprised 14 257 young women (15–24 y of age). We followed the Strengthening the Reporting of Observational Studies in Epidemiology guidelines in conducting this study. The data set is freely available to the public at https://dhsprogram.com/data/available-datasets.cfm (accessed on 11 December 2020).

### Definition of variables

#### Dependent variable

The dependent variable for the study was intended pregnancy, which arose from the question regarding whether women wanted their current pregnancy or not. It had three responses: then, later and not at all. Following the definition of unintended pregnancy as pregnancies that are wanted earlier or later than occurred (mistimed) or not wanted (unwanted),^13,14^ we coded the responses as 1=intended earlier or later and 0=unintended.

#### Explanatory variables

Based on the literature review,^3,4,5,14^ we considered 11 explanatory variables. These comprised age (15–19 y, 20–24 y), education level (no education, primary, secondary/higher), knowledge of contraceptive methods (knows no method, knows traditional, knows modern), marital status (never married, married, cohabiting, widowed/separated), employment status (not working, working), religion (Christian, Muslim, other), parity (zero, one, two, three or four or more births), intentions to use contraceptives (intend to use, does not intend to use), wealth index (poorest, poorer, middle, richer, richest), residence (rural, urban) and subregion (West Africa, East Africa, Central Africa, Southern Africa).

### Statistical analyses

The analyses began with the computation of intended pregnancy among young women in the 29 countries in SSA. We then appended the datasets. After appending, we presented the weighted sociodemographic characteristics of the young women. Subsequently, a chi-square test was used to describe the relationship between the independent variables and intended pregnancy. The final step involved a multivariable binary logistic regression using three models. Model I showed the association between the individual-level factors and intended pregnancy, model II showed the relationship between the contextual-level factors and intended pregnancy and model III showed the relationship between both the individual- and contextual-level variables and intended pregnancy. The results of the multivariable binary logistic regression models were presented as adjusted odds ratios (aORs) with their corresponding 95% confidence intervals (CIs) signifying precision. The analyses were carried out with Stata version 13.0 (StataCorp, College Station, TX, USA) with inherent sample weight applied. The survey command (svy) was also used to account for the complex sampling design of the survey.

### Ethical approval

The DHS report that ethical clearances were obtained from the ethics committee of ORC Macro as well as the ethics boards of partner organisations such as the Ministries of Health of the various countries. The DHS follow the standards for ensuring the protection of respondents’ privacy. Inner City Fund International ensures that the survey complies with the US Department of Health and Human Services’ regulations for the respect of human subjects. Since this was a secondary analysis, no further ethical approval was required because the datasets are available for download in the public domain. Further information about DHS data usage and ethical standards are available at http://goo.gl/ny8T6X.

## Results

### Prevalence of intended pregnancy among young women in SSA

**Figure 1.**
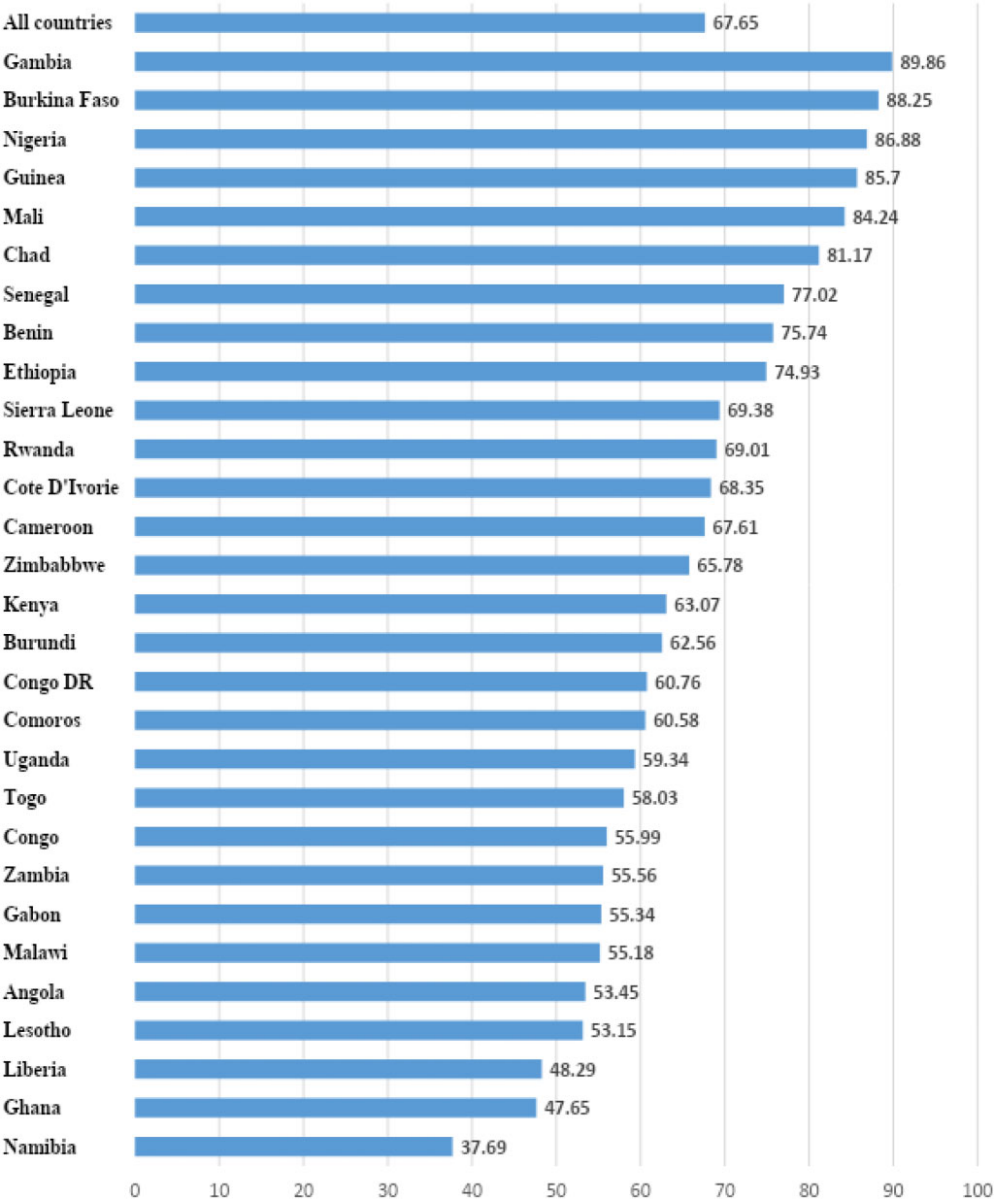
Prevalence of intended pregnancy among young women in SSA.

The prevalence of intended pregnancies among young women (15–24 y) in the 29 SSA countries considered in this study was 67.7%, with the highest and lowest prevalence in Gambia (89.9%) and Namibia (37.7%), respectively (Figure 1).

### Distribution of intended pregnancy across the individual- and contextual-level characteristics of young women

All the individual- and contextual-level variables had significant associations with intended pregnancy in the chi-square test, but variations were observed across the categories of the variables. The highest prevalence of intended pregnancy was observed among respondents ages 20–24 y (71.1%), those with no formal education (82.5%), those who knew no method of birth control (83.7%) and those who were married (79.4%). Intended pregnancy was also highest among respondents who were working (70.5%), Muslims (80.0%), those with one birth (70.0%), those who had no intention to use contraceptives (77.0%), the poorest adolescent girls and young women (70.7%), rural residents (70.3%) and those in West Africa (78.5%) (Table 1).

**Table 1.** Intended pregnancy among young women by explanatory variables (N=14 257 weighted)

Variables	Weighted N	Weighted %	Intended pregnancy, %	p-Value
Age (years)				<0.001
15–19	5267	36.9	61.8	
20–24	8989	63.1	71.1	
Education				<0.001
No education	4293	30.1	82.5	
Primary	5057	35.5	62.9	
Secondary/higher	4906	34.4	59.6	
Knowledge of contraceptives				<0.001
None	961	6.7	83.7	
Traditional	77	0.5	78.0	
Modern	13 220	92.7	66.4	
Marital status				<0.001
Never married	2253	15.8	31.1	
Married	8523	59.8	79.4	
Cohabiting	3082	21.6	64.5	
Widowed/separated	398	2.82.7	47.0	
Employment status				<0.001
Not working	5709	40.1	63.4	
Working	8548	59.9	70.5	
Religion				<0.001
Christian	8673	60.8	60.8	
Muslim	4979	34.9	80.0	
Other	605	4.3	64.0	
Parity				<0.001
0	6265	43.9	65.5	
1	4512	31.7	70.0	
2	2293	16.1	69.0	
3	890	6.2	68.0	
≥4	297	2.1	65.1	
Intentions to use contraceptives				<0.001
Intend to use	9046	63.5	62.2	
Does not intend to use	5211	36.5	77.0	
Wealth index				0.001
Poorest	3051	21.4	70.7	
Poorer	3275	23.0	68.4	
Middle	2952	20.7	67.4	
Richer	2832	19.9	65.5	
Richest	2147	15.1	65.4	
Residence				<0.001
Urban	4662	32.7	62.2	
Rural	9595	67.3	70.3	
Subregion				<0.001
West Africa	5241	36.8	78.5	
East Africa	5383	37.8	61.3	
Central Africa	3329	23.3	63.5	
Southern Africa	303	2.1	39.9	

* p-Values are from chi-square analysis.

### Results of multivariable logistic regression on determinants of intended pregnancy among young women in SSA

In terms of the individual-level factors, the likelihood of intended pregnancy was higher among young women ages 20–24 y compared with those ages 15–19 y (aOR 1.74 [95% CI 1.59 to 1.92]). Married young women were more likely to have intended pregnancies compared with never-married young women (aOR 7.17 [95% CI 6.36 to 8.07]). The likelihood of intended pregnancy was higher among young women who were working compared with those who were not working (aOR 1.29 [95% CI 1.19 to 1.40]) and Muslims compared with Christians (aOR 1.27 [95% CI 1.13 to 1.42]). Conversely, the likelihood of intended pregnancy was lower among young women who had knowledge of modern contraceptives compared with those who had no knowledge (aOR 0.64 [95% CI 0.53 to 0.78]), those with a secondary/higher level of education compared with those with no formal education (aOR 0.57 [95% CI 0.51 to 0.65]) and those with four or more births compared with those with no births (aOR 0.24 [95% CI 0.18 to 0.31]). With the contextual-level factors, lower odds of intended pregnancy were observed among young women in the richer wealth quintile compared with those in the poorest wealth quintile (aOR 0.81 [95% CI 0.71 to 0.92]) and those who lived in Southern Africa compared with those who lived in West Africa (aOR 0.67 [95% CI 0.50 to 0.88]) (Table 2).

**Table 2.** Multivariable logistic regression results on determinants of intended pregnancy among young women in SSA

Variables	Model I, aOR (95% CI)	Model II, aOR (95% CI)	Model III, aOR (95% CI)
Age (years)			
15–19	1		1
20–24	1.72*** (1.56 to 1.89)		1.74*** (1.59 to 1.92)
Education			
None	1		1
Primary	0.56*** (0.50 to 0.63)		0.60*** (0.54 to 0.68)
Secondary/higher	0.53*** (0.47 to 0.60)		0.57*** (0.51 to 0.65)
Knowledge of contraceptives			
None	1		1
Traditional	0.87 (0.48 to 1.58)		0.83 (0.48 to 1.45)
Modern	0.66*** (0.55 to 0.79)		0.64*** (0.53 to 0.78)
Marital status			
Never married	1		1
Married	7.16*** (6.34 to 8.01)		7.17*** (6.36 to 8.07)
Cohabiting	4.19*** (3.69 to 4.75)		4.24*** (3.73 to 4.83)
Widowed/separated	2.05*** (1.62 to 2.58)		2.11*** (1.67 to 2.67)
Employment status			
Not working	1		1
Working	1.34*** (1.23 to 1.45)		1.29*** (1.19 to 1.40)
Religion			
Christian	1		1
Muslim	1.40*** (1.26 to 1.54)		1.27*** (1.13 to 1.42)
Other	1.04 (0.86 to 1.26)		0.96 (0.79 to 1.17)
Parity			
0	1		1
1	0.63*** (0.57 to 0.70)		0.63*** (0.57 to 0.70)
2	0.42*** (0.37 to 0.48)		0.42*** (0.36 to 0.47)
3	0.34*** (0.28 to 0.40)		0.33*** (0.28 to 0.40)
≥4	0.24*** (0.18 to 0.31)		0.24*** (0.18 to 0.31)
Intentions to use contraceptives			
Intend to use	0.61*** (0.56 to 0.67)		0.63*** (0.57 to 0.69)
Does not intend to use	1		1
Wealth index			
Poorest		1	1
Poorer		0.87** (0.79 to 0.97)	0.89 (0.80 to 1.00)
Middle		0.86** (0.77 to 0.96)	0.89* (0.79 to 1.00)
Richer		0.88* (0.78 to 0.99)	0.81** (0.71 to 0.92)
Richest		1.06 (0.92 to 1.22)	0.90 (0.77 to 1.06)
Residence			
Urban		0.70*** (0.64 to 0.77)	0.97 (0.88 to 1.08)
Rural		1	1
Subregion			
West Africa		1	1
East Africa		0.43*** (0.40 to 0.47)	0.76*** (0.68 to 0.85)
Central Africa		0.52*** (0.47 to 0.57)	0.83** (0.73 to 0.94)
Southern Africa		0.18*** (0.14 to 0.23)	0.67*** (0.50 to 0.88)
N	14 257	14 257	14 257
Pseudo-R^2^	0.152	0.032	0.154

Exponentiated coefficients. 1=reference category.

*p<0.05, **p<0.01, ***p<0.001.

## Discussion

Several studies have suggested that most pregnancies among young women are unplanned or unintended.^17,18^ However, some of these pregnancies are intended. Therefore, given the dearth of knowledge about the determinants of intended pregnancies among young women in SSA, we examined the prevalence and determinants of intended pregnancies among young women in SSA using pooled data from the DHS of 29 countries. The prevalence of intended pregnancies was 67.7%. Our findings also suggest that Gambia has the highest prevalence of intended pregnancies among young women, with about 8 of 10 pregnancies being intended, while Namibia had the lowest prevalence of intended pregnancies among young women.

Beyond the numbers, our results indicate that the likelihood of intended pregnancy was lower among young women who had knowledge of modern contraceptives compared with those with no knowledge, thus implying that knowledge of modern contraceptives (e.g. condoms, implants, injectables etc.) is a protective factors against intended pregnancy among young women in SSA. This result corroborates previous studies in Ethiopia,^19,20^ Ghana^21^ and South Africa.^22^ A possible justification for our findings could be that young women's knowledge of modern contraceptives may translate into actual use of contraceptives. Consequently, this prevents pregnancy in these women, subsequently reducing their odds of intended pregnancies (or pregnancy in general).

In this study we found that formal education is a protective factor against intended pregnancies in SSA. Specifically, it is clear from our study that young women with a secondary/higher level of education were less likely to have an intended pregnancy compared with those with no formal education. This is consistent with previous study by Faisal-Cury et al.^23^ that showed a lower level of education independently associated with planned pregnancies among adolescents. The findings may be explained by the fact that young women who have a secondary/higher education are privy to a wide range of accurate information and knowledge about modern contraceptives and therefore are more likely to use them.^21^ Thus they are less likely to get pregnant at a young age. Faisal-Cury et al.^23^ also postulate it is very likely that young women with no formal education may have a greater desire for childbirth in their adolescence, thereby resulting in higher odds of intended pregnancy among those with no formal education. This indicates strongly that keeping young women in school and providing them with a formal education could be an effective strategy to drastically reduce intended pregnancies at young ages in SSA.

Moreover, our findings suggest that the likelihood of intended pregnancy is higher among married young women in SSA compared with their counterparts who are not married. The result mirrors studies conducted in some parts of SSA, including Kenya,^24^ that showed marital status is significantly associated with intended pregnancies, i.e. married young women are more likely to experience intended pregnancy. A possible justification for this result could be that in the African context, high value is placed on childbirth and in most SSA countries the accepted norm is to give birth in marriage and not out of wedlock. Therefore young women who are married are expected by their husbands, their family and even their communities to give birth. Hence married young women do not consider pregnancy in marriage as unintended. In addition, a similar study conducted in Nigeria^25^ and Ghana^26^ showed that husbands often discourage their wives from using modern contraceptives. As such, married young women refrain from using family planning or contraceptives on the assumption that no pregnancy within marriage is unintended. Our findings also underscore the importance of ending adolescent marriages in SSA if indeed the region intends to reduce intended pregnancies among young women and its associated morbidity and mortality risks.

We also found age to be significantly associated with the odds of reporting a pregnancy as intended in SSA, as the likelihood of intended pregnancy was higher among young women ages 20–24 y compared with those ages 15–19 y. This result is analogous to that in the literature.^24,25^ This could be attributed to the ages of these groups. The younger age group (15–19 y) is still in the period of adolescence, a period where most of them initiate sex despite their sexual inexperience and ignorance about the correct use of modern contraceptives.^25^ As such, they are less likely to have an intended pregnancy. However, for the older age group (20–24 y), most of them have gained knowledge and experience about the need to protect themselves from unintended pregnancy and are therefore more likely to get pregnant only when they intend to. Most young women also get married at this age and would therefore want to have children. We also noticed that intended pregnancies are more likely to be reported among young women who are working. Similar inferences could be made from a study conducted by Ameyaw et al.^2^ that indicated women who are working are less likely to experience unintended pregnancies but rather report higher odds of intended pregnancies. Women who are working are usually overwhelmed with their work demands and are therefore conscious about their pregnancies.

Our findings also suggest a significant association between religion and intended pregnancies among young women in SSA. Muslim young women were most likely to have intended pregnancies compared with those who are Christian. Related studies in Nigeria^26^ and Tanzania^27^ support our findings that Muslim young women are most likely to have intended pregnancies. However, this result is inconsistent with Rassi et al.,^28^ who found no significant association between religion and intended pregnancies. Possibly this result could be explained from the perspective that Muslim women may marry early in their reproductive years and, given that pregnancy and childbirth are expected right after marriage in SSA, it becomes a reason for them to perceive their pregnancies as intended.

With respect to the contextual issues, our study found that young women in the richer wealth quintile had lower odds of intended pregnancies compared with those in the poorer wealth quintile. Similar findings have been reported by Lamina^29^ that suggest the odds of intended pregnancies decrease as wealth increases. A plausible justification for our findings may be that women in the richer wealth quintile may be exposed to the media as well as sexual and reproductive health information and services, thereby limiting their risks of intended pregnancies. Moreover, women who are employed tend to be empowered in every facet of their lives and thus have control over their sexuality,^2^ resulting in a delay in pregnancy.

Approximately 61.8% of pregnancies among girls 15–19 y old are intended. This has implications when it comes to public health strategies. It is known that unintended pregnancies are more likely to end up as unsafe abortions in this age group in most LMICs.^1,6^ However, attaining the SDGs on good health and well-being (goal 3), quality education (goal 4) and decent work and economic growth (goal 8) will require that pregnancies among girls 15–19 y old, both intended and unintended, be reduced.^12^ Indeed, in some countries like Mali and Niger, >20% of women give birth before the age of 16 y.^30^ Culturally sensitive strategies are therefore needed, especially in settings where early marriages and adolescent pregnancies are encouraged or considered normal. Indeed, interventions generally treating adolescent pregnancies as unintended might not be effective in reducing adolescent pregnancy rates. A clear research agenda is therefore required to determine specific interventions for intended and unintended pregnancies. Drawing from the socio-ecological model,^31^ individual (knowledge and attitudes, economic status), interpersonal (peers, communication climate with parents on sexuality issues, partner responsibility), community (religious considerations, social media exposure, community attitudes towards early adolescent pregnancy, cultural desirability of children), organizational (fees to access safe abortion care services, abortion-associated stigma, trust) and policy (abortion laws, conscientious objection) factors play specific roles in driving intended and unintended pregnancies. An understanding of the predisposing factors at each level, as well as how these factors interact with each other, is needed in planning specific interventions.

Finally, we found that although the prevalence of intended pregnancies among young women in SSA is high, it differed between geographical boundaries. Our study indicates that there are lower odds of intended pregnancy among young women who live in Southern Africa compared with those who live in West Africa. This result brings to the fore that there cannot be a one-size-fits-all strategy or approach targeted at reducing intended pregnancies in SSA, since the dynamics differ across the various subregions within SSA. Hence there is the need for country-specific studies to develop a deeper understanding of the geospatial variations in the prevalence and determinants of intended pregnancies in SSA. Robust qualitative studies geared towards understanding the reasons why young women (especially adolescents) intentionally get pregnant are needed. Understanding the commonalities and differences regarding the determinants of intended and unintended pregnancies is of utmost relevance in setting up holistic interventions responsive to the specificities of both.

### Strengths and limitations

Our study's strength lies in the use of a nationally representative dataset from 29 countries in SSA under the DHS programme. This reinforces the generalizability of our study findings to the general population of young women. Moreover, the use of both individual and contextual variables to test significant associations with intended pregnancy ensured the rigor of our analyses and models, thereby ensuring the reliability and replicability of the study to other populations. Notwithstanding the apparent strengths of our study, we caution that our findings be interpreted in the light of some limitations. Given that the dataset used employed cross-sectional designs, it limits the possibility of causal inferences primarily because of the snapshot nature of such designs. The data used in this study is retrospective self-reporting data and, as such, it may be biased by the mother's perception or feeling about pregnancy, which may change over time.

### Practical implications

Our study has practical implications. First, our findings draw attention to the need to invest heavily in the education of young women in SSA, as it is a significantly protective factor associated with intended pregnancy. When more girls receive formal education, they are likely to delay pregnancy and that will further reduce the odds of intended pregnancy in SSA. Also, the results of our study indicate that the odds of intended pregnancy vary across the various subregions of SSA. This highlights an important point that policies and strategies targeted at reducing intended pregnancies among young women in SSA must consider contextual issues in order to be viable. Overall, our study suggests that in order to achieve the SDGs as well as prevent the morbidity and mortality risks associated with pregnancy among young women, it is imperative to invest in the education of young women, provide them with sustainable jobs and eradicate poverty.

## Conclusions

To reduce intended pregnancies in SSA countries such as Gambia, Burkina Faso and Nigeria, there is the need for government and non-governmental organisations to recalibrate current and past interventions, including increasing formal education for women, poverty alleviation programmes and job creation, including skill building. These interventions have to be sensitive to the cultural realities of each setting, especially concerning early marriages and womanhood.

## Data Availability

The dataset is freely available to the public at https://dhsprogram.com/data/available-datasets.cfm.
